# Mental health status among physiotherapists. A cross-sectional study in Tunisia

**DOI:** 10.1192/j.eurpsy.2025.2291

**Published:** 2025-08-26

**Authors:** A. Aloui, M. Bouhoula, I. Kacem, E. Toulgui, A. Chouchane, M. Maoua, A. Brahem, H. Kalboussi, O. El Maalel, W. Ouanes, S. Jemni, S. Chatti

**Affiliations:** 1 Occupational Medecine department, University Hospital of Farhat Hached, Sousse, Tunisia; 2 Faculty of Medecine Ibn El Jazzar, University of Sousse, 4000 Sousse, Tunisia; 3 Research laboratory LR 19SP03, Study of the risks and prospects for the prevention of non-communicable diseases in the workplace; 4 Physical Medecine and Rehabilitation Department, University Hospital of Sahloul, Sousse, Tunisia, Tunisia

## Abstract

**Introduction:**

In the high-stress world of healthcare, the intertwined relationship mental health and the life quality is pivotal.

Prioritizing healthcare professionals’ mental health and nurturing a positive safety climate are essential to enhancing overall job satisfaction.

**Objectives:**

Evaluate the mental health status of the physiotherapists.

**Methods:**

his is a cross-sectional study among Physiotherapists (PTs) working in the city of Sousse. A self-administrated questionnaire was distributed either manually or via e-mail to collect information about the personal and occupational history of participants. The mentel health was assesed by the the mental component score (MCS-12) of the the Short Form Survey SF-12.

**Results:**

A total of 93 questionnaires were collected. The PTs were 35±8 years old on average. Women made up 65.6% of the PTs. Sixty-six (71.7%) and sixty-two (67.4%) did not provide any medical or surgical history respectively and the majority of the population (54.5%) had a normal BMI. Among all respondents, nineteen (20.4%) PTs were regular smokers and an alcoholic beverage was consumed by 14 (15.7%) PTs. The sport was the common hobby of PTs (53.8%). In this group of PTs, 37 (40.2%) worked in the public sector, and 55 (59.8%) worked in the private sector. The mean seniority was 11.9±7.8 years and the average workweek for the population was 42.42 hours. The mean MCS-12 score was 43.94 (9.05 SD). The findings showed that 35 PTs (37.6%) had MCS-12 scores below the standard value. Male PTs showed slightly higher MCS-12 means than female PTs with a modest difference between PTs working in the public and private sectors. PTs who had more than 15 years of work experience scored the lowest MCS-12 mean 41.40 (SD 9.94) which is a score underneath the reference value indicating in this case low mental health functioning among PTs.

**Conclusions:**

This study showed that Tunisian PTs had low mental health statuts highlighting the need for approaches to improve interventions that effectively enhance well-being, retention, and sustainability of practitioners, and thus the care delivered, in the healthcare system

**Disclosure of Interest:**

None Declared

